# Associations of chronic kidney disease with recurrent stroke in patients with intracerebral haemorrhage

**DOI:** 10.1093/esj/aakaf007

**Published:** 2026-01-01

**Authors:** Philip S Nash, Simon Fandler-Höfler, Gareth Ambler, Hatice Ozkan, Larysa Panteleienko, Rom Mendel, Wenpeng Zhang, Lena Obergottsberger, Linda Fabisch, Gerit Wünsch, Hans Rolf Jäger, Christian Enzinger, David C Wheeler, Robert J Simister, Thomas Gattringer, David J Werring

**Affiliations:** UCL Stroke Research Centre, Department of Applied Neuroscience and Stroke, University College London Queen Square Institute of Neurology, London, United Kingdom; Comprehensive Stroke Service, National Hospital for Neurology and Neurosurgery, University College London Hospitals NHS Trust, London, United Kingdom; UCL Stroke Research Centre, Department of Applied Neuroscience and Stroke, University College London Queen Square Institute of Neurology, London, United Kingdom; Department of Neurology, Medical University of Graz, Graz, Austria; Department of Statistical Science, University College London, London, United Kingdom; UCL Stroke Research Centre, Department of Applied Neuroscience and Stroke, University College London Queen Square Institute of Neurology, London, United Kingdom; Comprehensive Stroke Service, National Hospital for Neurology and Neurosurgery, University College London Hospitals NHS Trust, London, United Kingdom; UCL Stroke Research Centre, Department of Applied Neuroscience and Stroke, University College London Queen Square Institute of Neurology, London, United Kingdom; UCL Stroke Research Centre, Department of Applied Neuroscience and Stroke, University College London Queen Square Institute of Neurology, London, United Kingdom; UCL Stroke Research Centre, Department of Applied Neuroscience and Stroke, University College London Queen Square Institute of Neurology, London, United Kingdom; Department of Neurology, Medical University of Graz, Graz, Austria; Department of Neurology, Medical University of Graz, Graz, Austria; Institute for Medical Informatics, Statistics and Documentation, Medical University of Graz, Graz, Austria; Lysholm Department of Neuroradiology and the Neuroradiological Academic Unit, Department of Applied Neuroscience and Stroke, UCL Institute of Neurology, London, United Kingdom; Department of Neurology, Medical University of Graz, Graz, Austria; Department of Renal Medicine, University College London, London, United Kingdom; Comprehensive Stroke Service, National Hospital for Neurology and Neurosurgery, University College London Hospitals NHS Trust, London, United Kingdom; Department of Neurology, Medical University of Graz, Graz, Austria; UCL Stroke Research Centre, Department of Applied Neuroscience and Stroke, University College London Queen Square Institute of Neurology, London, United Kingdom; Comprehensive Stroke Service, National Hospital for Neurology and Neurosurgery, University College London Hospitals NHS Trust, London, United Kingdom

**Keywords:** intracerebral haemorrhage, chronic kidney disease, recurrent stroke, intracerebral haemorrhage aetiology, ischaemic stroke aetiology, cerebral small vessel disease

## Abstract

**Background:**

Chronic kidney disease (CKD) is a frequent comorbidity of patients with intracerebral haemorrhage (ICH) and is associated with more severe cerebral small vessel disease. Whether CKD is associated with recurrent stroke after ICH is unknown.

**Patients and methods:**

We conducted a retrospective cohort study of 2 comprehensive stroke centres, collecting data from consecutive patients with ICH. Patients with secondary causes of ICH were excluded. We defined CKD according to Kidney Disease: Improving Global Outcomes definitions, namely 2 measurements of estimated glomerular filtration rate (eGFR) < 60 mL/min/1.73 m^2^ ≥ 3 months apart. The primary outcome was time to any stroke (recurrent ICH or ischaemic stroke), investigated using Cox regression adjusted for age, sex and comorbidities. Outcomes were confirmed by neuroimaging review.

**Results:**

A total of 1062 patients (mean age 68 ± 14 years, 45% female) with ICH were included, 239 with CKD. Over a median (IQR) follow-up of 2.3 (0.7–5.0) years, there was a higher rate of any stroke in the CKD group, 8.4 (95% CI, 6.2–11.1) events per 100 person-years vs 4.4 (3.6–5.3) events in the group with normal eGFR (adjusted hazard ratio [aHR] 1.75: 95% CI, 1.23–2.50, *P* = .002). CKD was also independently associated with both recurrent ICH (aHR 1.81: 95% CI, 1.15–2.85) and ischaemic stroke (aHR 1.78: 95% CI, 1.06–3.01).

**Conclusion:**

Patients with ICH and CKD are at increased risk of recurrent ICH and ischaemic stroke compared to those with normal eGFR. Further research is needed into this high-risk patient group to identify new prevention treatments.

## Graphical Abstract

**Graphical Abstract:**
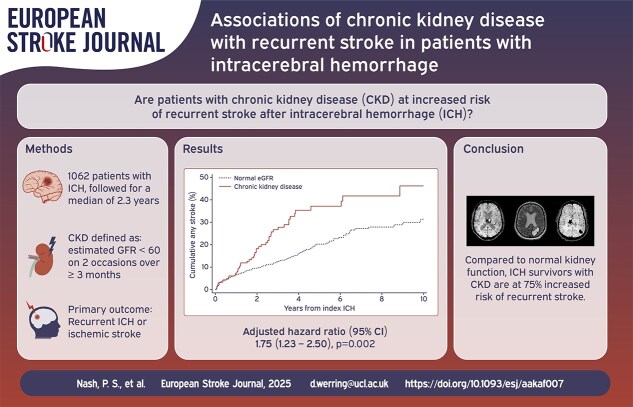

## Introduction

Intracerebral haemorrhage (ICH) is a health condition of global importance.^1^ In 2019 it caused more than 3 million strokes, accounting for a higher burden of disability than ischaemic stroke.^2^ A meta-analysis of population-based studies reported survival rates of 46% and 29% at 1 and 5 years, respectively.^3^ The incidence and prognosis worldwide are static despite improvements in primary and secondary stroke prevention.^4^^,^^5^ Cerebral small vessel disease (cSVD) causes approximately 80% of spontaneous ICH^6^ and is an important cause of ischaemic stroke and dementia.^7^ Its pathogenesis is incompletely understood, and further research is needed to develop treatments.^8^

Chronic kidney disease (CKD) is associated with adverse cardiovascular events,^9^ and its global prevalence is estimated to have increased by 29% since 1990, with mortality attributable to CKD increasing by 42%.^10^ It has previously been associated with both cSVD^11^ and ICH^12^ in community populations. A recent Global Burden of Diseases report found that number of disability-adjusted life-years attributed to impaired kidney function as a risk factor for stroke has been increasing from 1990 to 2019,^2^ and further study of this relationship should be a research priority. However, there are very few studies investigating clinical outcomes after ICH according to kidney function. Existing reports concern mainly functional outcome^13-16^ and mortality,^17^^,^^18^ and we are not aware of studies investigating CKD as a risk factor for recurrent stroke.

The majority of studies investigating CKD in stroke populations have used a single laboratory measurement as their definition of renal impairment. This approach is imprecise and does not account for bias in the results caused by acute kidney injury, which is frequently present in patients with acute conditions such as stroke.^19^^,^^20^ We previously showed that CKD, defined according to Kidney Disease: Improving Global Outcomes (KDIGO) criteria,^21^ is independently associated with mixed location cSVD and severe markers of arteriolosclerosis.^22^ Whether this translates into increased risk of recurrent stroke after ICH is not known.

Therefore, in this 2-centre study of consecutive patients with ICH, we aimed to determine whether CKD is associated with recurrent stroke, both recurrent ICH and ischaemic stroke, major adverse cardiovascular events (MACE), early mortality (within 21 days of onset) or all-cause mortality during follow-up.

## Patients and methods

This study is reported according to Strengthening the Reporting of Observational Studies in Epidemiology recommendations.

### Patient selection

The design of the SIGNAL (Stroke Investigation Group in North and Central London)—Graz 2-centre ICH cohort study has been described previously.^22^ Briefly, consecutive adult (>18 years) patients presenting to University College Hospital London (UCLH) or the University Hospital of the Medical University of Graz with imaging-confirmed non-traumatic ICH were selected for retrospective study. Patients from SIGNAL were only eligible if they also attended UCLH for long-term follow-up. Both centres performed magnetic resonance imaging (MRI) for all patients with ICH unless contraindicated. Key exclusion criteria were secondary causes of ICH such as arterio-venous malformations, tumours, cerebral venous sinus thrombosis and hemorrhagic transformation of ischaemic infarcts. Patients with convexity subarachnoid haemorrhage or pure intraventricular haemorrhage were also excluded.

The studies at each centre were approved by their corresponding local Research Ethics Committees (REC reference for UCLH 24/LO/0368, approval reference for Graz 32-265 ex 19/20). As the data were collected as part of routine clinical care and there were no interventions, the need for individual written consent was waived.

### CKD definition

CKD was defined according to the internationally accepted KDIGO recommendations,^21^ namely 2 blood samples with estimated glomerular filtration rate (eGFR) < 60 mL/min/1.73 m^2^. These definitions have been shown to accurately predict adverse cardiovascular outcomes and mortality in a large individual patient data meta-analysis including over 27 million participants.^23^ At UCLH blood results were extracted from the electronic health record manually by the same nephrologist (P.S.N.), and at Graz a digital data extract was reviewed, with missing values extracted manually from the health records. To be classified as having CKD, all patients had to have at least 2 blood tests > 3 months apart. Patients with acute kidney injury that resolved and otherwise no indication of renal impairment were classified into the normal kidney function group. To estimate eGFR, we used the 2009 CKDEPI equation^24^ for this European cohort, omitting the ethnicity coefficient, as recently recommended by the European Renal Association^25^ and the European Federation of Clinical Chemistry and Laboratory Medicine.^26^ All CKD diagnoses were validated by P.S.N.

### Clinical variables

Hypertension was defined as a pre-existing diagnosis in the medical records, the use of 1 or more antihypertensives prior to stroke onset, or if antihypertensives were initiated at hospital discharge. Hypercholesterolemia was defined as a known history of raised cholesterol or lipid-lowering medication use at baseline or discharge. Diabetes was defined according to medical history or an HbA1c > 6.5% on presentation.^27^ Ischaemic heart disease was defined according to previous history of myocardial infarction, unstable angina, percutaneous or surgical coronary revascularisation. Atrial fibrillation was defined as a known medical history or a new diagnosis on an electrocardiogram. Heart failure was diagnosed according to the medical records or using echocardiography during the inpatient visit. The use of antiplatelets or anticoagulants before ICH onset was recorded, as was current or previous smoking, excessive alcohol intake (>14 units per week) or any illegal drug use.

### Neuroimaging rating procedures

These have been previously described in detail.^22^ Briefly, ICH location was defined according to Cerebral Haemorrhage Anatomical RaTing inStrument,^28^ and small vessel disease markers were rated according to Standards for Reporting Vascular Changes on Neuroimaging 2 (STRIVE-2) consensus definitions.^29^ A modified version of the Microbleed Anatomical Rating Scale^30^ was used to define cerebral microbleeds (CMB), the simplified Fazekas Scale^31^ was used to rate white matter hyperintensities, and a widely used 5-point ordinal scale was used to rate enlarged perivascular spaces in the basal ganglia and centrum semiovale.^32^ ICH aetiology was determined using a modified version of the CLAS-ICH definition,^33^ as reported previously.^34^ Probable cerebral amyloid angiopathy (CAA) was diagnosed according to Boston 2.0 criteria,^35^ or the simplified Edinburgh criteria^36^ if MR was unavailable. Arteriolosclerosis (sometimes also referred to as nonamyloid, hypertensive or deep perforator arteriopathy) was diagnosed in non-lobar ICH when there were strictly deep or infratentorial CMBs or no CMB with evidence of other cSVD markers. Mixed location cSVD was diagnosed when both lobar and non-lobar hemorrhagic markers were present, either the index ICH or CMB, or in lobar ICH with other non-hemorrhagic deep cSVD markers. If there was no significant cSVD and no evidence of a secondary ICH cause on detailed neuroimaging assessment, including angiography (either CT or MR angiogram), patients were classified as cryptogenic ICH.

### Outcomes

Our pre-specified primary outcome was time to any stroke during follow-up, a composite of recurrent ICH and ischaemic stroke. Outcomes for all patients were determined by screening the electronic health records for both centres, and surrounding hospital care systems for Graz. Potential outcome events were validated through detailed case-by-case review, including neuroimaging confirmation for stroke events. Follow-up was censored on the date of the confirmed outcome event or the date of last clinical contact if there was no event. Recurrent ICH was classified as lobar, deep, brainstem or cerebellar location and ischaemic stroke events were classified according to TOAST criteria.^37^

Secondary outcomes included the individual components of the primary outcome, all-cause mortality, death within 21 days and MACEs (a composite of ICH, ischaemic stroke, myocardial infarction and transient ischaemic attack).

### Statistical analysis

The patient population was classified according to the presence or absence of CKD. Participant characteristics were described using descriptive statistics. Numerical variables were summarised using mean (SD) or median (IQR) depending on variable distributions, and compared using *t* tests or Mann–Whitney U tests as appropriate. Categorical variables were described using numbers (%) and compared using the χ-squared test.

The times to recurrent events were determined from the date of the index ICH. Event rates were compared using the log-rank test and illustrated using Kaplan–Meier plots. We assessed the proportional hazards assumption using log–log plots, and then fitted univariable and multivariable Cox regression models to investigate the risk of outcome events according to the presence or absence of CKD. The prespecified list of covariates included in the multivariable models was chosen according to biological plausibility and included age, sex, hypertension, diabetes, hyperlipidemia, atrial fibrillation, congestive heart failure, previous stroke or TIA, current or previous smoking and lobar location of the index ICH. These variables could all be associated with either ICH or cSVD risk based on existing evidence. We did an additional analysis including ICH aetiology in the regression models for those with MR available. Because of a high death rate in the CKD group, we did a competing risks analysis, with time to death as the competing risk. For the primary outcome, we did an additional analysis according to CKD severity, with severity categorised according to KDIGO recommendations^21^: stage 2 CKD—eGFR 60–90 (with historic evidence of eGFR < 60 and/or albuminuria on at least 2 occasions > 3 months apart); stage 3—eGFR 30–60 and stages 4 and 5—eGFR < 30. KDIGO stage 3a was merged with 3b, and 4 merged with 5 owing to small group sizes. To assess risk of death within 21 days of ICH onset, we fitted a logistic regression model with death within 21 days as the dependent variable and CKD, age, sex, hypertension, diabetes, heart failure, previous stroke or TIA, current or previous smoking, anticoagulation prior to ICH, log(ICH volume), and lobar index ICH as independent variables. ICH volume was transformed owing to skewedness. We adjusted for study site in all regression models using fixed effects. We imputed missing values for covariates using multiple imputation with chained equations (50 imputations). There were no missing outcome data or CKD data.

We performed subgroup analyses according to the location and aetiology of the index ICH. We fitted Cox proportional hazards models including interaction terms of CKD with: (1) location, lobar vs non-lobar; (2) aetiology, CAA vs non-CAA determined using either CT or MR based diagnostic criteria and (3) MR-determined ICH aetiology, classified as cryptogenic ICH, arteriolosclerosis, mixed cSVD or CAA. We adjusted for age, sex and study site in the subgroup analyses.

After independent peer review, we performed a sensitivity analysis where we excluded patients with CKD classified based on laboratory results from after the index ICH, to assess the impact of possible immortal time bias. We also performed supplementary analyses where did not exclude those with early mortality and measured the time to event analyses from after day 21 to the point of an event or censorship, to estimate the impact of any potential selection bias.

Statistical analyses were performed using STATA (version 18; StataCorp LLC).

## Results

**Figure 1:**
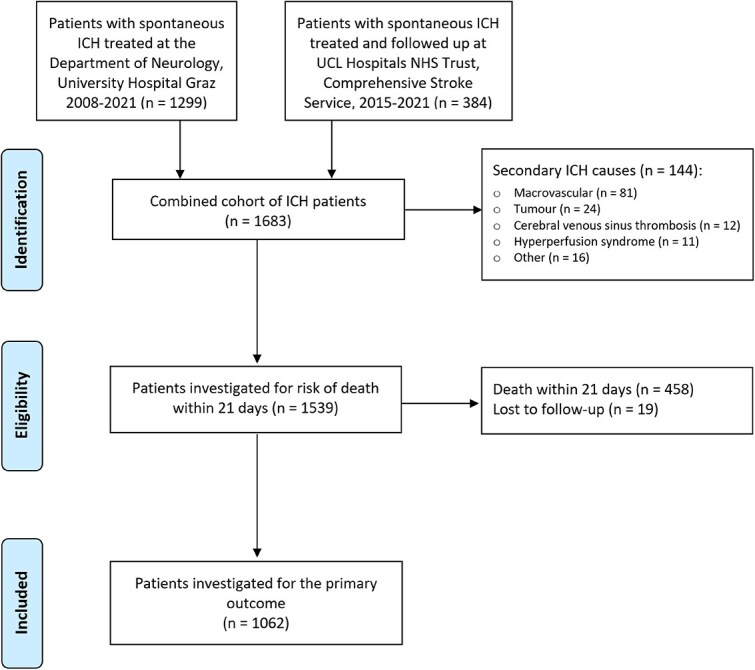
Study flowchart of patient selection. Abbreviation: ICH = intracerebral haemorrhage.

In total, 1683 patients were assessed for eligibility, of whom 144 were excluded for secondary causes of ICH, 458 died before 21 days and 19 were lost to follow-up, leaving 1062 patients for inclusion in our primary outcome analysis, 239 with CKD. A total of 82% of patients with CKD were diagnosed based on either 2 blood tests before onset, or 1 at onset and 1 > 3 months before. Consequently, 18% were classified based on bloods during the index hospitalisation and >3 months afterwards, as shown in Figure S1. Patient flow through the study is shown in Figure 1. Baseline characteristics of patients included in the primary analysis are shown in Table 1. Characteristics of the study population investigated for early mortality are shown in Table S1. Compared to the group of patients with normal kidney function, the CKD group was older with higher rates of cardiovascular risk factors such as hypertension, diabetes, hypercholesterolemia, ischaemic heart disease and previous stroke.

**Table 1:** Baseline characteristics of included participants.

	**All patients (*n* = 1062)**	**Normal eGFR (*n* = 823)**	CKD **(*n* = 239)**	***P*-value**
**Clinical variables**				
Age (years); mean (SD)	68.4 (14.0)	67.0 (13.5)	73.2 (14.5)	<.001
Female sex; no. (%)	481 (45.2%)	373 (45.3%)	108 (45.2%)	.983
Hypertension	854 (80.4%)	646 (78.5%)	208 (87.0%)	.003
Diabetes	207 (19.5%)	125 (15.2%)	82 (34.3%)	<.001
Hyperlipidemia	116 (10.9%)	86 (10.4%)	30 (12.6%)	.359
Ischemic heart disease	101 (9.5%)	61 (7.4%)	40 (16.7%)	<.001
Atrial fibrillation	162 (15.2%)	102 (12.4%)	60 (25.1%)	<.001
Heart failure	32 (3.0%)	21 (2.5%)	11 (4.6%)	.102
Previous stroke or TIA	149 (14.0%)	98 (11.9%)	51 (21.3%)	<.001
Current or previous smoking^a^	130 (12.6%)	109 (13.7%)	21 (8.9%)	.050
Alcohol excess	102 (9.6%)	88 (10.7%)	14 (5.9%)	.025
Illegal drug use	14 (1.3%)	12 (1.5%)	2 (0.8%)	.456
Anticoagulation before ICH	137 (14.5%)	82 (11.2%)	55 (25.7%)	<.001
Antiplatelets before ICH	219 (22.0%)	156 (20.2%)	63 (28.3%)	.011
Systolic BP on arrival	176.4 (34.1)	175.8 (33.8)	178.6 (34.9)	.312
eGFR; mean (SD)	72.4 (23.5)	78.0 (20.7)	53.2 (22.6)	
Length index hospital stay; days, median (IQR)	18 (9–32)	18 (9–35)	16 (10–27)	.124
Follow-up duration; years, median (IQR)	2.3 (0.7–5.0)	2.5 (0.8–5.3)	1.7 (0.4–4.1)	
**Index ICH characteristics**				
Volume (ml); median (IQR)	9.1 (3.2–23.7)	9.7 (3.5–25.3)	7.7 (2.6–19.3)	.007
Location				.004
Lobar	402 (37.9%)	326 (39.6%)	76 (31.8%)	
Deep	525 (49.4%)	400 (48.6%)	125 (52.3%)	
Cerebellar	85 (8.0%)	64 (7.8%)	21 (8.8%)	
Brainstem	47 (4.4%)	33 (4.0%)	14 (5.9%)	
Multifocal	3 (0.3%)	0 (0%)	3 (1.3%)	
Intraventricular extension	353 (33.2%)	287 (34.9%)	66 (27.6%)	.036
Subarachnoid extension	190 (19.2%)	158 (20.7%)	32 (14.1%)	.027
Finger-like projections	141 (14.2%)	110 (14.4%)	31 (13.7%)	.784
CT probable CAA diagnosis^b^	74 (7.5%)	63 (8.2%)	11 (4.8%)	.087
MRI-based ICH aetiology^c^				.002
Cryptogenic ICH	104 (15.5%)	93 (16.8%)	11 (9.4%)	
Arteriolosclerosis	163 (24.3%)	134 (24.2%)	29 (24.8%)	
Mixed cSVD	258 (38.5%)	197 (35.6%)	61 (52.1%)	
Probable CAA	145 (21.6%)	129 (23.3%)	16 (13.7%)	

^a^Using simplified Edinburgh criteria.

^b^Data on smoking status missing for 28 patients.

^c^MRI unavailable for 392 participants.

Abbreviations: BP = blood pressure; eGFR = estimated glomerular filtration rate; ICH = intracerebral haemorrhage; cSVD = cerebral small vessel disease; CAA = cerebral amyloid angiopathy.

### Primary outcome—any stroke during follow-up

**Figure 2:**
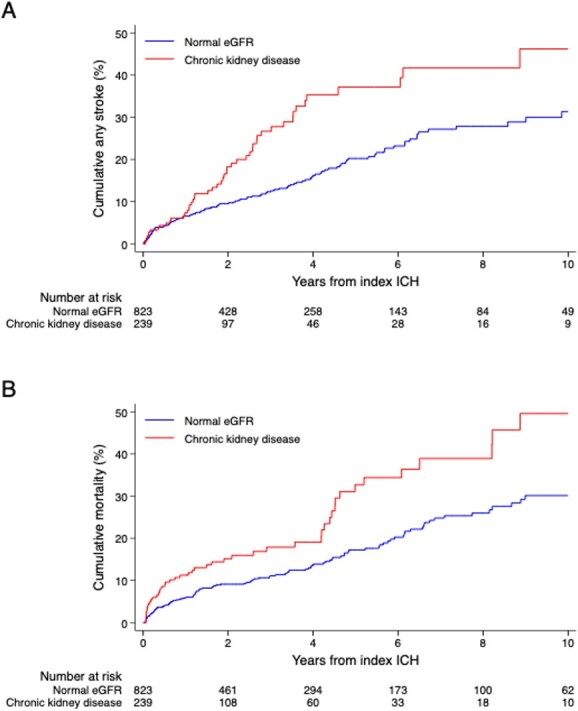
Event rate plots for (A) any stroke and (B) all-cause mortality according to CKD. Abbreviations: eGFR = estimated glomerular filtration rate; ICH, intracerebral haemorrhage; CKD = chronic kidney disease.

Over a median (IQR) follow-up of 2.3 (0.7–5.0) years, there were 182 composite stroke events, a rate of 5.1 per 100 person-years of follow-up. Compared to the normal eGFR group, there were significantly more stroke events in the CKD group, with an event rate (95% CI) of 8.4 (6.2–11.1) vs 4.4 (3.6–5.3), log-rank *P* < .001. After adjustment for age, sex, index ICH location and comorbidities, CKD was independently associated with an increased risk of recurrent stroke, adjusted HR (aHR) 1.75: 95% CI, 1.23–2.50, *P* = .002 (Table 2, Figure 2). Full Cox regression models are shown in Table S2 in the supplementary materials. In the analysis of CKD severity, the point estimates for stage 2 (aHR 1.73: 95% CI, 1.03–2.91) and stage 3 (aHR 1.70: 95% CI, 1.09–2.66) CKD were similar, but with increased risk for stages 4 and 5 (aHR 2.15: 95% CI, 0.85–5.43, *P* for trend = .021, Table 2). There were insufficient patients receiving dialysis or kidney transplant recipients to do a detailed analysis, but out of 17 patients, 5 had a primary outcome event (29%), the highest rate for all CKD severities.

**Table 2:** Outcome events according to kidney function.

	**Number of events**	**Follow-up (person-years)**	**1-year risk (95% CI)**	**3-year risk (95% CI)**	**5-year risk (95% CI)**	**Recurrence rate per 100 person-Years (95% CI)**	**Adjusted HR (95% CI)**
**Any stroke**							
All patients	166 (15.6%)	3247	7 (5–9)	15 (13–18)	24 (20–28)	5.1 (4.4–6.0)	-
Normal eGFR	117 (14.2 %)	2664	7 (5–9)	12 (10–15)	20 (17–25)	4.4 (3.6–5.3)	Ref.
CKD	49 (20.5%)	584	7 (4–12)	27 (19–35)	37 (28–47)	8.4 (6.2–11.1)	1.75 (1.23–2.50)
							1.54 (0.97–2.44)^b^
Stage 2 CKD^a^ (*n* = 78)	17 (21.8%)	238	9 (4–20)	26 (15–41)	36 (23–53)	7.2 (4.2–11.4)	1.73 (1.03–2.91)
Stage 3 CKD (*n* = 131)	27 (20.6)	295	5 (2–12)	27 (18–39)	38 (26–53)	9.2 (6.0–13.3)	1.70 (1.09–2.66)
Stage 4 and 5 CKD (*n* = 30)	5 (16.7)	51	11 (3–39)	27 (11–59)	42 (18–77)	9.7 (3.2–22.7)	2.15 (0.85–5.43)
**Recurrent ICH**							
All patients	106 (10.0%)	3419	4 (3–6)	9 (7–11)	15 (12–19)	3.1 (2.5–3.7)	-
Normal eGFR	77 (9.4%)	2798	4 (3–6)	8 (6–10)	14 (11–17)	2.8 (2.1–3.4)	Ref.
CKD	29 (12.1%)	621	5 (2–9)	13 (8–20)	21 (14–30)	4.7 (3.1–6.7)	1.81 (1.15–2.85)
							1.89 (1.05–3.40)^b^
**Ischemic stroke**							
All patients	73 (6.9%)	3420	3 (2–4)	7 (5–9)	10 (8–13)	2.1 (1.6–2.7)	-
Normal eGFR	49 (6.0%)	2812	3 (2–4)	5 (4–7)	8 (6–11)	1.7 (1.3–2.3)	Ref.
CKD	24 (10.0%)	608	3 (1–7)	15 (10–23)	22 (14–32)	3.9 (2.5–5.9)	1.78 (1.06–3.01)
**Major cardiovascular events**							
All patients	193 (18.2%)	3167	8 (6–10)	17 (15–21)	27 (24–31)	6.1 (5.3–7.0)	-
Normal eGFR	138 (16.8%)	2616	7 (6–9)	15 (12–18)	23 (20–28)	5.3 (4.4–6.2)	Ref.
CKD	55 (23.0%)	551	9 (5–14)	29 (22–37)	43 (34–54)	10.0 (7.5–13.0)	1.61 (1.15–2.25)
**All-cause mortality**							
All patients	167 (15.7%)	3602	7 (6–9)	13 (10–15)	20 (17–24)	4.6 (4.0–5.4)	-
Normal eGFR	117 (14.2%)	2957	6 (4–8)	11 (9–14)	17 (14–21)	4.0 (3.3–4.7)	Ref.
CKD	50 (20.9%)	646	11 (8–16)	18 (13–25)	33 (24–43)	7.7 (5.7–10.2)	1.45 (1.02–2.06)

^a^Stage 2 CKD—eGFR 60-90 (with historic evidence of eGFR < 60 and/or albuminuria on at least 2 occasions > 3 months apart); stage 3—eGFR 30-60 and stages 4 and 5—eGFR < 30.

^b^Model additionally adjusted for index ICH aetiology.

Abbreviations: eGFR = estimated glomerular filtration rate; CKD = chronic kidney disease; ICH = intracerebral haemorrhage.

All Cox regression models adjusted for age, sex, hypertension, diabetes, hyperlipidemia, atrial fibrillation, heart failure, previous ischemic stroke or transient ischemic attack, current or previous smoking and lobar location of index ICH; clustering adjusted for using fixed effects.

In the competing risks regression analysis, using the same independent variables in the model with time to any stroke as the dependent variable and time to death as the competing risk, there was no significant change to the estimates (aHR for CKD vs normal kidney function 1.61: 95% CI, 1.12–2.32).

In the sensitivity analysis excluding patients with CKD classified based on blood taken > 3 months after ICH onset the estimates were consistent with our primary analysis, aHR 1.95 (95% CI, 1.33–2.87). The supplementary analyses including all patients with available follow-up (*n* = 1520) and measuring time to event from after day 21 were also consistent with our primary analysis. In the Cox proportional hazards models and competing risks regression models the aHR (95% CI) for CKD vs normal eGFR was 1.79 (1.25–2.57) and 1.64 (1.13–2.38), respectively. There were just 3 primary outcome events before day 21.

### Secondary outcomes

The rate of recurrent ICH was higher in the CKD group at 4.7 per 100 person-years (95% CI, 3.1–6.7) compared with the normal eGFR group at 2.8 (2.1–3.4). This was also the case for ischaemic stroke, 3.9 (2.5–5.9) vs 1.7 (1.3–2.3). Event rates and multivariable HRs of the secondary outcomes are shown in Table 2 and illustrated in Figure S2. After adjusting for age, sex and covariates, compared to normal eGFR, CKD was independently associated with the risk of recurrent ICH (aHR 1.81: 95% CI, 1.15–2.85), ischaemic stroke (aHR 1.78: 95% CI, 1.06–3.01), major cardiovascular events (aHR 1.61: 95% CI, 1.15–2.25), and all-cause mortality during follow-up (aHR 1.45: 95% CI, 1.02–2.06, Figure 2).

### Early mortality

In total, 458 patients died within 21 days of the index ICH (29.8%), 319 (27.6%) with normal kidney function and 139 (36.3%) with CKD. Compared to normal kidney function, CKD was independently associated with early mortality in a logistic regression model adjusted for age, sex, hypertension, diabetes, heart failure, previous stroke or TIA, anticoagulation at baseline, systolic blood pressure on presentation, log(ICH volume), lobar ICH location and study centre, adjusted odds ratio (aOR) 1.60, (95% CI, 1.14–2.25). Full details are shown in Table S3 and illustrated in Figure S3 in the supplementary materials. In a logistic regression model investigating early mortality according to CKD severity, adjusted for the same potential confounders, compared to normal kidney function, there was no increased risk of death for CKD stage 2 (aOR 0.88: 95% CI), but a stepwise increased risk for CKD stage 3 (aOR 1.54: 95% CI, 1.02–2.32) and CKD stages 4 and 5 (aOR 4.26: 95% CI, 2.07–8.78).

### Subgroup analysis

**Figure 3:**
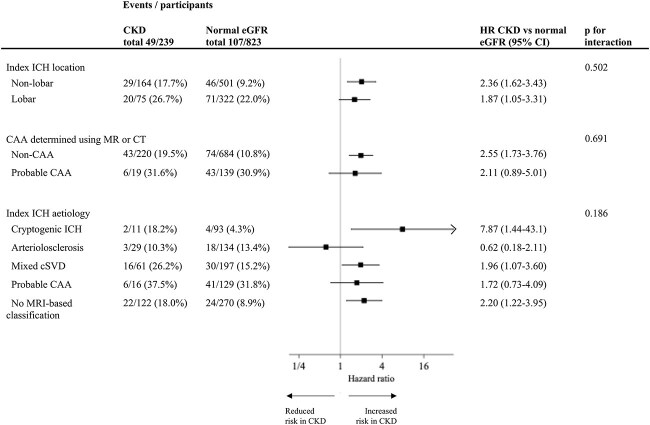
Subgroup analysis showing risk of any stroke according to kidney function, location and aetiology of the index ICH. Abbreviations: CKD = chronic kidney disease; eGFR = estimated glomerular filtration rate; ICH = intracerebral haemorrhage; cSVD = cerebral small vessel disease; CAA = cerebral amyloid angiopathy. Interaction Cox regression models adjusted for age, sex and study site using fixed effects.

Outcome event rates for any stroke according to CKD in different subgroups of index ICH location and aetiology are shown in Figure 3. In Cox regression models, adjusted for age, sex and study centre, there was no significant heterogeneity of the increased risk of any stroke according to CKD, apart from a slightly reduced risk in those with arteriolosclerosis as the index ICH aetiology.

### Aetiology of recurrent stroke events

**Figure 4:**
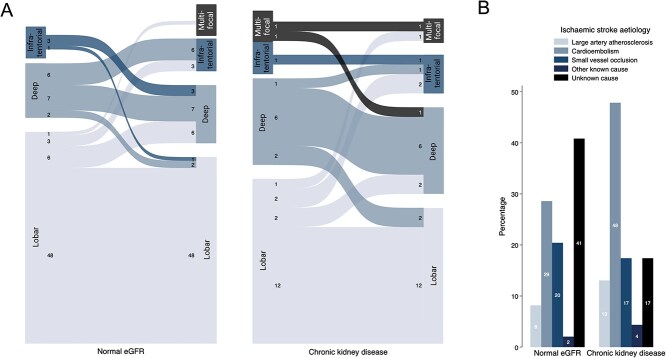
(A) ICH locations at index and recurrence and (B) ischemic stroke aetiology according to kidney function. Abbreviations: eGFR = estimated glomerular filtration rate; ICH = intracerebral haemorrhage. In the 4A index, ICH location is on the left and recurrent ICH location on the right for each study group created in BioRender. Nash, P. (2025). https://BioRender.com/jqlyrc.

Compared to normal eGFR, there was a wider range of locations of recurrent ICH and a wider range of ischaemic stroke etiologies (Table S4, Figure 4). There was also a higher rate of recurrent ICH at a different location from the index event. There were nearly double the rates of ischaemic strokes caused by large artery atherosclerosis (13% vs 8%) and cardioembolism (48% vs 29%) in the CKD group.

## Discussion

In this large 2-centre observational study, our major finding was that compared to normal kidney function, CKD was independently associated with a 75% increased risk of any stroke during a median duration of 2.3 years of follow-up in patients with ICH. We are not aware of previous reports describing this association, which was consistent across index ICH locations and aetiologies after adjustment for confounding factors. The increased stroke risk associated with CKD was particularly marked for those with a non-lobar index ICH, with a nearly 2.5-fold increased risk. We also found that CKD was independently associated with recurrent ICH, ischaemic stroke, MACEs, death within 21 days, and all-cause mortality during follow-up.

Previous investigations of outcomes after ICH according to kidney function are limited. Post-hoc analyses of the multinational INTERACT-2^16^ and ATACH-2^13^ trials found independent associations of eGFR < 60 mL/min/1.73 m^2^ with disability or death at 90 days. A small prospective Japanese cohort study had similar findings,^14^ but a multicentre UK prospective cohort study did not find an independent association.^15^ A Chinese registry study with a similar size to the present study (*n* = 1268) found an independent association of renal dysfunction (eGFR < 60) with in-hospital mortality.^17^ Another small study found an independent association of renal dysfunction with mortality at 1 year.^18^ However, this was only present for the eGFR < 45 subgroup. Another study with a similar sample size (*n* = 1076) to the present analysis found an independent association of CKD (defined as eGFR < 60) with poor functional outcome and mortality at 1 year.^38^ However, these studies did not report recurrent stroke as an outcome or use the KDIGO-recommended definition of CKD.

A recent large individual patient data meta-analysis including data from over 11,000 patients with ischaemic stroke or transient ischaemic attack^39^ demonstrated an independent association of impaired kidney function (eGFR < 60) with recurrent stroke, with an HR of 1.33 (95% CI, 1.14–1.56). Compared to eGFR > 60, there was a higher rate of ischaemic stroke in the eGFR < 60 group, but not intracranial haemorrhage. We are not aware of any similar studies in ICH populations. In the present study, we found substantially increased rates of both ischemic stroke and recurrent ICH in patients with CKD, which might be explained in part by the higher baseline risk of recurrent ICH in a population of patients with ICH who are likely to have more severe cSVD (the commonest cause of ICH) at baseline. To our knowledge, this is the first study reporting associations of recurrent stroke after ICH according to CKD.

There are several possible mechanisms explaining the increased risk of both recurrent ICH and ischaemic stroke in patients with CKD. Of note, those with CKD had a wider range of both index and recurrent ICH locations (Figure 4). For 31% of the patients with CKD and recurrent ICH, the recurrence was in a different location (lobar vs non-lobar), more than those with normal kidney function (17% of recurrences in a different location). By contrast, 62% of those with normal eGFR and ICH recurrence in our study had lobar ICH at both index presentation and recurrence, and this proportion increased to 93% for those with CAA. This is in keeping with recent evidence showing that CAA-related ICH recurs in temporal and spatial clusters.^40^ The variation in ICH locations at index presentation and recurrence in CKD could be related to the more diffuse and severe cSVD in the CKD group in our population, of arteriolosclerosis phenotype, as previously reported.^22^ As the highest risk of recurrent stroke for the CKD group was in those with non-lobar index ICH, this suggests that CKD might contribute to the pathogenesis of more severe arteriolosclerosis. In addition to the high rates of known cSVD risk factors in the CKD group, this more widespread and advanced microvascular damage could be related to the build-up of uremic protein breakdown products which accumulate in CKD and can be toxic to vascular endothelium.^41^^,^^42^ The graded increased risk of recurrent stroke for those with severe (grades 4–5) CKD is also supportive of these potential mechanisms of recurrence in CKD. ICH recurrence in the normal kidney function group was primarily driven by CAA, which had a high rate of early recurrence (8.3% vs 0.6% for non-CAA) and only had a prevalence of 13.7% in the CKD group. This is the main reason behind the similar rates of stroke recurrence in the CKD and normal kidney function groups for the first year of follow-up, after which the rate of recurrence increases in the CKD group, driven mainly by mechanisms unrelated to CAA. We previously reported this low rate of CAA in CKD, including a particularly low rate of cortical superficial siderosis (2.5%).^22^ This finding is supported by a case series of 40 autopsies of patients with CKD, where arteriolosclerosis was found in 73% of specimens and just 1 case of CAA.^43^ Nevertheless, this relationship is understudied and should be investigated in future research.

For ischaemic stroke, there were 66% higher rates of cardioembolic and carotid stenosis-related stroke in the CKD group compared to normal kidney function, reflecting the higher burden of comorbidities such as atrial fibrillation and coronary atherosclerosis in CKD in our population. Higher rates of atrial fibrillation and carotid stenosis have been extensively reported in CKD,^44-46^ and proposed direct CKD-related mechanisms include cardiac myocyte toxicity caused by indoxyl sulphate which accumulates in CKD,^47^ or increased vascular calcification and arterial stiffness related to impaired calcium-phosphate homeostasis in CKD and subsequent secondary hyperparathyroidism.^48^

The strengths of this study include: its large sample size; the inclusion of consecutive patients limiting selection bias; recruitment from 2 centres increasing the generalisability of the findings and the long duration of follow-up. Our definition of CKD is more precise than most studies investigating stroke, and our centres have a have a high utilisation of MRI (63% in this study population) which increases the accuracy of ICH aetiology classification. Our study also has limitations which we acknowledge. Its observational design means that despite multivariable adjustments the results are subject to residual confounding. Although we had detailed past medical history data for study participants, we did not have access to markers of the severity of each comorbidity. To thoroughly investigate risk of recurrent stroke, we excluded those with early mortality, and a high proportion of these patients had CKD (37% compared to 25% in the entire sample). This introduces possible selection bias as these potentially frailer patients with CKD have been excluded from the primary outcome analysis. However, our supplementary analyses estimate that the impact of any selection bias is small, and there were only 3 primary outcome events before day 21. Another limitation is that we did not have data available on the resumption of antithrombotic medication after ICH, so we were unable to assess whether restarting these treatments carries more risk in those with CKD. Finally, we did not have systematically collected data on albuminuria, so some participants meeting diagnostic criteria for CKD based on albuminuria will have been misclassified.

## Conclusion

In this large cohort study of patients with ICH, compared to normal kidney function, CKD was independently associated with increased risk of any stroke, recurrent ICH, ischaemic stroke, major cardiovascular events and both short- and long-term mortality. Future research should focus on the development of tailored treatments for this high-risk patient group.

## Supplementary Material

aakaf007_SIGNAL_Graz_ICH_cohort_study_revision_supplement

## Data Availability

To facilitate the replication of procedures and outcomes, inquiries for anonymised data that were not included in the article will be considered from appropriately qualified researchers. A data-sharing agreement must be put in place before any data are shared. Written proposals will be assessed by the senior members of the SIGNAL-Graz collaboration.
