# Supplementary material for: Associations of chronic kidney disease with recurrent stroke in patients with intracerebral haemorrhage
Source: Eur Stroke J. 2026 Jan 1;11(1):aakaf007. doi: 10.1093/esj/aakaf007 (PMC12866628; doi:10.1093/esj/aakaf007)
Supplement: aakaf007_SIGNAL_Graz_ICH_cohort_study_revision_supplement [file aakaf007_signal_graz_ich_cohort_study_revision_supplement.docx]

**Supplementary Material**

**Supplementary Table S1.** Baseline characteristics of the analysis population for early mortality according to chronic kidney disease presence

|  | Missing | All patients (n=1539) | Normal eGFR (n=1156) | Chronic kidney disease (n=383) | p-value |
| --- | --- | --- | --- | --- | --- |
| **Clinical variables** |  |  |  |  |  |
| Age (years); mean(SD) | 0 (0%) | 70.5 (14.0) | 69.0 (13.7) | 75.1 (13.8) | <0.001 |
| Female sex; no.(%) | 0 (0%) | 737 (47.9%) | 548 (47.4%) | 189 (49.3%) | 0.510 |
| Hypertension | 6 (0.4%) | 1178 (76.8%) | 861 (74.7%) | 317 (83.2%) | <0.001 |
| Diabetes | 5 (0.3%) | 291 (19.0%) | 171 (14.8%) | 120 (31.5%) | <0.001 |
| Hyperlipidemia | 7 (0.5%) | 187 (12.2%) | 132 (11.5%) | 55 (14.5%) | 0.119 |
| Ischaemic heart disease | 7 (0.5%) | 177 (11.6%) | 98 (8.5%) | 79 (20.8%) | <0.001 |
| Atrial fibrillation | 5 (0.3%) | 287 (18.7%) | 168 (14.6%) | 119 (31.2%) | <0.001 |
| Heart failure | 5 (0.3%) | 64 (4.2%) | 32 (2.8%) | 32 (8.4%) | <0.001 |
| Previous Stroke or TIA | 0 (0%) | 229 (14.9%) | 152 (13.1%) | 77 (20.1%) | <0.001 |
| Current or previous smoking | 45 (2.9%) | 173 (11.6%) | 134 (12.0%) | 39 (10.3%) | 0.386 |
| Alcohol Excess | 12 (0.8%) | 134 (8.8%) | 115 (10.0%) | 19 (5.0%) | 0.003 |
| Illegal drug use | 12 (0.8%) | 22 (1.4%) | 20 (1.7%) | 2 (0.5%) | 0.084 |
| Anticoagulation | 174 (11.3%) | 263 (19.3%) | 163 (16.0%) | 100 (28.8%) | <0.001 |
| Antiplatelet | 126 (8.2%) | 353 (25.0%) | 244 (23.0%) | 109 (30.8%) | 0.004 |
| Systolic BP on arrival | 424 (27.6%) | 177.1 (34.8) | 176.4 (34.7) | 179.5 (35.2) | 0.193 |
| eGFR; mean (SD) |  | 71.0 (24.0) | 77.8 (20.5) | 50.5 (22.0) | <0.001 |
| **Index ICH characteristics** |  |  |  |  |  |
| Volume (ml); median (IQR) | 98 (6.4%) | 15.4 (4.9 to 43.5) | 16.0 (5.1 to 45.4) | 14.1 (4.1 to 42.0) | 0.142 |
| Location | 17 (1.1%) |  |  |  | 0.022 |
| Lobar |  | 615 (40.4%) | 481 (42.1%) | 134 (35.3%) |  |
| Deep |  | 714 (46.9%) | 530 (46.4%) | 184 (48.4%) |  |
| Cerebellar |  | 113 (7.4%) | 79 (6.9%) | 34 (8.9%) |  |
| Brainstem |  | 73 (4.8%) | 49 (4.3%) | 24 (6.3%) |  |
| Multifocal |  | 7 (0.5%) | 3 (0.3%) | 4 (1.1%) |  |
| Intraventricular extension | 0 (0%) | 661 (42.9%) | 498 (43.1%) | 163 (42.6%) | 0.858 |
| Subarachnoid extension | 92 (6.0%) | 363 (25.1%) | 285 (26.4%) | 78 (21.3%) | 0.054 |
| Finger-like projections | 91 (5.9%) | 243 (16.8%) | 188 (17.4%) | 55 (15.0%) | 0.299 |
| Index ICH aetiology* |  |  |  |  | 0.002 |
| Cryptogenic ICH |  | 109 (15.2%) | 97 (16.7%) | 12 (9.0%) |  |
| Arteriolosclerosis |  | 173 (24.2%) | 139 (23.9%) | 34 (25.4%) |  |
| Mixed cSVD |  | 279 (39.0%) | 210 (36.1%) | 69 (51.5%) |  |
| Probable CAA |  | 154 (21.5%) | 135 (23.2%) | 19 (14.2%) |  |

Abbreviations: BP = blood pressure; eGFR = estimated glomerular filtration rate; ICH = intracerebral hemorrhage; cSVD = cerebral small vessel disease; CAA = cerebral amyloid angiopathy

*MRI unavailable for 824 participants

**Supplementary Table S2.** Univariable and multivariable Cox regression analysis for risk of the primary outcome, any stroke, according to chronic kidney disease

|  | Univariable |  | Multivariable |  |
| --- | --- | --- | --- | --- |
|  |  |  |  |  |
| **Predictor** | **HR (95% CI)** | **p-value** | **HR (95% CI)** | **p-value** |
| Chronic kidney disease | 1.825 (1.306 to 2.551) | <0.001 | 1.749 (1.226 to 2.497) | 0.002 |
| Age | 1.027 (1.015 to 1.040) | <0.001 | 1.021 (1.007 to 1.035) | 0.003 |
| Female sex | 0.968 (0.712 to 1.316) | 0.836 | 0.936 (0.682 to 1.284) | 0.680 |
| Hypertension | 0.934 (0.641 to 1.363) | 0.725 | 1.063 (0.718 to 1.575) | 0.760 |
| Diabetes | 0.996 (0.674 to 1.473) | 0.986 | 0.944 (0.629 to 1.416) | 0.780 |
| Hyperlipidemia | 1.629 (1.028 to 2.581) | 0.038 | 1.279 (0.766 to 2.137) | 0.347 |
| Atrial fibrillation | 1.466 (0.997 to 2.156) | 0.052 | 1.120 (0.747 to 1.678) | 0.584 |
| Heart failure | 1.732 (0.811 to 3.696) | 0.156 | 1.736 (0.797 to 3.777) | 0.165 |
| Previous Stroke or TIA | 1.674 (1.138 to 2.464) | 0.009 | 1.331 (0.885 to 2.003) | 0.170 |
| Current or previous smoking | 1.157 (0.737 to 1.814) | 0.526 | 1.326 (0.828 to 2.123) | 0.241 |
| Lobar index ICH | 2.203 (1.621 to 2.995) | <0.001 | 2.266 (1.650 to 3.111) | <0.001 |

Abbreviations: ICH = intracerebral hemorrhage; TIA = transient ischemic attack

**Supplementary Table S3.** Univariable and multivariable logistic regression analysis for risk of death within 21 days according to chronic kidney disease

|  | Univariable |  | Multivariable |  |
| --- | --- | --- | --- | --- |
|  |  |  |  |  |
| **Predictor** | **OR (95% CI)** | **p-value** | **OR (95% CI)** | **p-value** |
| Chronic kidney disease | 1.495 (1.170 to 1.910) | 0.001 | 1.600 (1.140 to 2.247) | 0.007 |
| Age | 1.041 (1.032 to 1.051) | <0.001 | 1.042 (1.029 to 1.055) | <0.001 |
| Female sex | 1.360 (1.092 to 1.693) | 0.006 | 1.150 (0.856 to 1.545) | 0.353 |
| Systolic BP on arrival | 1.002 (0.998 to 1.006) | 0.234 | 1.003 (0.998 to 1.008) | 0.214 |
| Hypertension | 0.545 (0.425 to 0.699) | <0.001 | 0.515 (0.365 to 0.727) | <0.001 |
| Diabetes | 0.883 (0.665 to 1.174) | 0.394 | 0.814 (0.553 to 1.199) | 0.297 |
| Heart failure | 2.494 (1.508 to 4.123) | <0.001 | 1.511 (0.781 to 2.920) | 0.220 |
| Previous Stroke or TIA | 1.151 (0.852 to 1.556) | 0.360 | 1.009 (0.672 to 1.514) | 0.967 |
| Current or previous smoking | 0.658 (0.452 to 0.958) | 0.029 | 1.254 (0.766 to 2.052) | 0.369 |
| Anticoagulation | 2.537 (1.921 to 3.351) | <0.001 | 1.748 (1.192 to 2.562) | 0.004 |
| Log(ICH volume) | 2.892 (2.545 to 3.287) | <0.001 | 3.236 (2.778 to 3.768) | <0.001 |
| Lobar index ICH | 1.422 (1.136 to 1.778) | 0.002 | 0.453 (0.325 to 0.631) | <0.001 |

Abbreviations: ICH = intracerebral hemorrhage; TIA = transient ischemic attack

**Supplementary Table S4.** Etiology of recurrent stroke events

|  | Normal eGFR (n=823) | Chronic kidney disease (n=239) | All (n=1062) |
| --- | --- | --- | --- |
| Total recurrent ICH | 77 (9.4%) | 29 (12.1%) | 106 (10.0%) |
| Recurrent ICH location |  |  |  |
| Lobar | 51 (66.2%) | 14 (48.3%) | 65 (61.3%) |
| Deep | 16 (20.8%) | 9 (31.0%) | 25 (23.6%) |
| Cerebellar | 6 (7.8%) | 1 (3.4%) | 7 (6.6%) |
| Brainstem | 3 (3.9%) | 3 (10.3%) | 6 (5.7%) |
| Multifocal | 1 (1.3%) | 2 (6.9%) | 3 (2.8%) |
| Recurrence location compared to index |  |  |  |
| Lobar index and recurrence | 48 (62.3%) | 12 (41.4%) | 60 (56.6%) |
| Lobar index; non-lobar recurrence | 10 (13.0%) | 7 (24.1%) | 14 (13.2%) |
| Non-lobar index and recurrence | 17 (22.1%) | 8 (27.6%) | 27 (25.5%) |
| Non-lobar index; lobar recurrence | 3 (3.9%) | 2 (6.9%) | 5 (4.7%) |
| Ischemic stroke during follow-up | 49 (6.0%) | 24 (10.0%) | 73 (6.9%) |
| Ischemic stroke classification |  |  |  |
| Large artery atherosclerosis | 4 (8.2%) | 3 (13.0%) | 7 (9.7%) |
| Cardioembolism | 14 (28.6%) | 11 (47.8%) | 25 (34.7%) |
| Small vessel occlusion | 10 (20.4%) | 4 (17.4%) | 14 (19.4%) |
| Other known cause | 1 (2.0%) | 1 (4.3%) | 2 (2.8%) |
| Unknown cause | 20 (40.8%) | 4 (17.4%) | 24 (33.3%) |

eGFR, estimated glomerular filtration rate

**Supplementary Figure S1.** Timeline of chronic kidney diagnoses

**Supplementary Figure S2.** (A) Recurrent intracerebral haemorrhage, (B) ischaemic stroke and (C) major adverse cardiovascular events according to chronic kidney disease

Abbreviations: eGFR = estimated glomerular filtration rate; ICH = intracerebral hemorrhage; MACE = major adverse cardiovascular events

**Supplementary Figure S3.** Cumulative mortality before 21 days according to chronic kidney disease

 Abbreviations: eGFR = estimated glomerular filtration rate; ICH = intracerebral hemorrhage
